# Cas rare d’un rhabdomyosarcome du col: à propos d’un cas avec revue de la littérature

**DOI:** 10.11604/pamj.2016.25.166.8629

**Published:** 2016-11-16

**Authors:** Hamza Samlali, Hassan Jouhadi, Hicham Attar, Souha Sahraoui, Abdellatif Benider

**Affiliations:** 1 Centre Mohamed VI des Traitements des Cancers CHU IBN Rochd Casablanca; 2 Laboratoire d’Anatomo-Pathologie Moulay Idriss Casablanca

**Keywords:** Rabdomyosarcome, col utérin, botryoide, Rhabdomyosarcoma, uterine cervix, botryoid

## Abstract

Le rhabdomyosarcome du col utérin fait partie des types histologiques rares des cancers du col, qu'on observe généralement chez la jeune fille ou la femme en période d'activité générale. La stratégie thérapeutique se base sur l'association des trois modalités thérapeutiques (chimiothérapie-radiothérapie-chirurgie) vu l'agressivité de la maladie. Nous rapporterons ainsi un cas de rhabdomyosarcome du col utérin chez une fille de 20 ans. Il s'agit d'une patiente âgée de 20 ans, rapportant comme antécédents pathologique particulier la notion d'infection génitale à répétition. Le premier signe rapporté était des métrorragies abondantes avec à l'examen la présence d'une masse cervicale en grappe de raisin. La biopsie était en faveur d'un rhabdomyosarcome du col. Le bilan d'extension montrait une masse localement avancée sans métastase. La patiente a reçu 5 cures de VAC avec une régression du processus tumoral de 90%. Patiente fut opérée et a bénéficié d'une hystérectomie sans conservation des annexes. Puis elle a bénéficié d'une radiothérapie postopératoire sur le pelvis. Le recul était de 13 mois, la patiente est toujours en rémission complète. Le RMS du col utérin est une tumeur rare qui survit essentiellement chez la jeune fille. L'extension est surtout locorégionale. Le traitement consiste en un geste chirurgical allant d'un éventuel traitement conservateur jusqu'au traitement radical associé à une chimiothérapie péri-opératoire. La place de la radiothérapie demeure imprécise.

## Abstract (en)

Rhabdomyosarcoma of the uterine cervix is a rare histological type of cervical cancer, occurring commonly in young girls or sexually active women. Given the aggressiveness of the disease, therapeutic strategy is based on the combination of the three treatment modalities (chemotherapy – radiation therapy-surgery). We report the case of a 20-year old patient with rhabdomyosarcoma of the uterine cervix. The patient had a personal history of recurrent genital infections. Patient’s first symptom was profuse metrorrhagias associated with the presence of a mass like “a bunch of grapes”. Biopsy was in favor of a rhabdomyosarcoma of the uterine cervix. E Extension assessment showed locally advanced mass without metastasis. The patient underwent 5 administrations of VAC chemotherapy, exhibiting tumor regression of 90%. She underwent hysterectomy without adnexal conservation. Then she received postoperative pelvic radiation therapy. At 13-months’ follow-up the patient was still in complete remission. Rhabdomyosarcoma of the uterine cervix is a rare tumor that develops most often in young girls. It mainly shows locoregional extension. Treatment is based on surgery including conservative treatment as well as radical treatment associated with perioperative chemotherapy. The role of radiation therapy remains poorly defined.

## Introduction

Le rhabdomyosarcome du col fait partie des types histologiques rares des cancers du col, qu'on observe généralement chez la jeune fille ou la femme en période d'activité générale. Une centaine de cas sont décrits dans la littérature, les principales caractéristiques qui en sortent sont tout d'abord un aspect macroscopique particulier dit en « grappe de raisin », puis un haut potentiel métastatique contrairement aux carcinomes épidermoides du col à extension locorégionale. La stratégie thérapeutique se base sur l'association des trois modalités thérapeutiques (chimiothérapie-radiothérapie-chirurgie) vu l'agressivité de la maladie. La possibilité d'un traitement conservateur reste possible dans le cadre d'une prise en charge multidisciplinaire. Nous présentons ainsi l'observation d'une jeune fille de 20 ans suivie pour rhabdomyosarcome du col. Cette observation sera commentée par la suite par une revue de la littérature.

## Patient et observation

**Figure 1:**
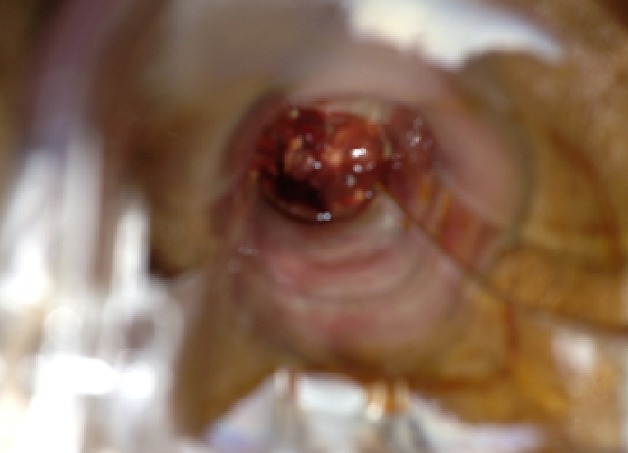
Masse cervicale en grappe de raisin sur examen sous speculum

**Figure 2:**
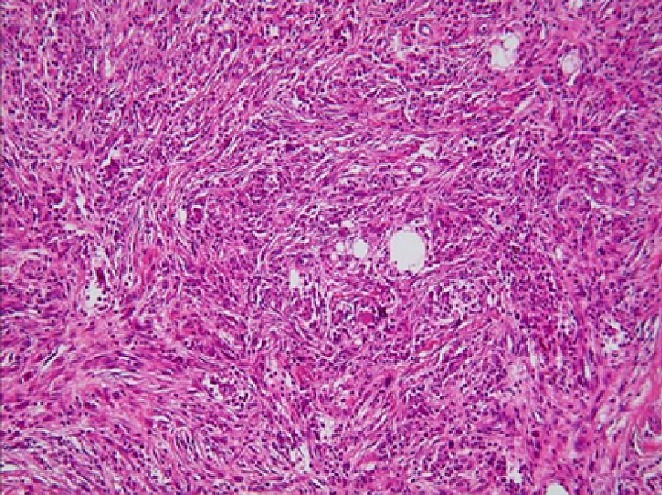
Prolifération de grandes cellules atypiques fusiformes à cytoplasme éosinophile en faveur d’un rhabdomyosarcome du col

**Figure 3:**
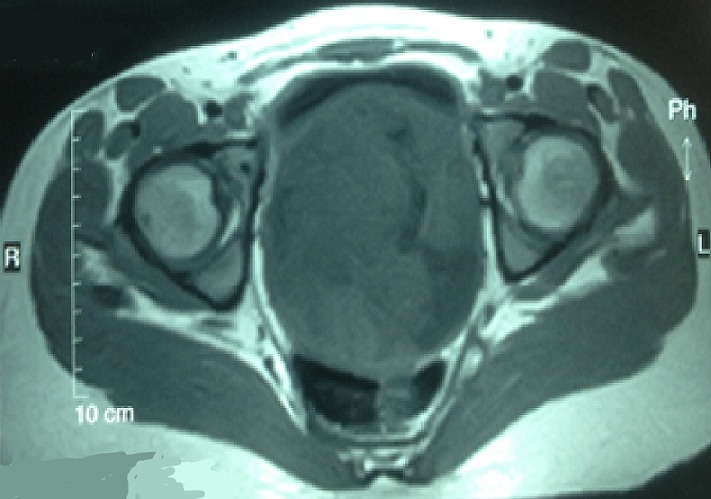
Coupe transversale de l’IRM abdomino-pelvienne en séquence t1 montrant la masse pelvienne

**Figure 4:**
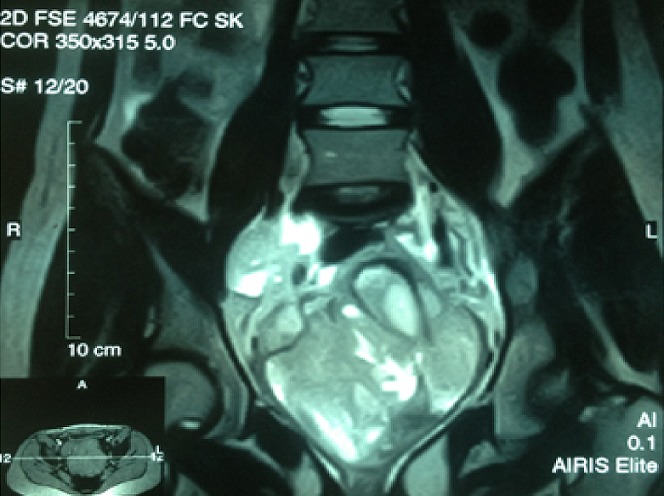
Coupe coronale de l’IRM abdomino-pelvienne en séquence t2 montrant la masse pelvienne localement avancé

**Figure 5:**
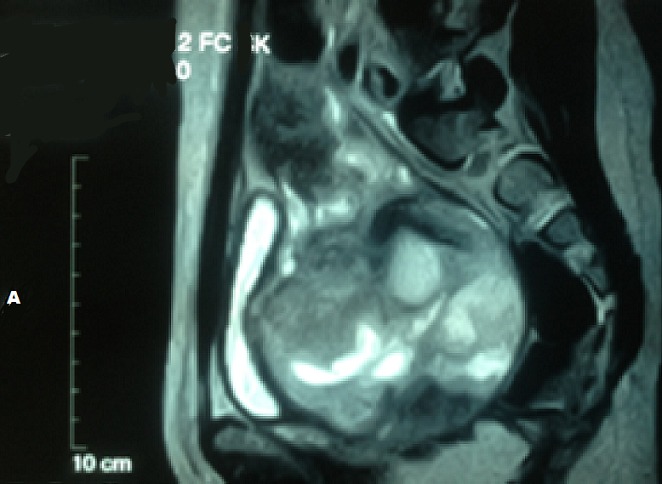
Coupe sagittale de l’IRM abdomino-pelvienne en séquence t2 montrant la masse pelvienne localement avancé

Il s'agit d'une fille de 20 ans présentant comme antécédent la notion d'infections gynécologiques à répétition. Cette patiente consultait initialement pour des métrorragies post-coïtales révélant un polype accouché par le col dont l'examen histologique ne montrait pas d'anomalie. La patiente fut perdue de vue et se présente à la suite de l'aggravation de la métrorragie devenant alors spontanée associé à une grossesse de 8SA. L'examen gynécologique montrait à l'inspection sous spéculum un processus tumoral polyploïde détruisant le col en grappe de raisin, le toucher vaginal rapportait une tumeur de 90*70 cm atteignant les 2/3 supérieur du vagin friable et hémorragique (Figure 1). Le toucher rectal montrait une énorme masse centro-pelvienne comprimant les 2 paramètres. L'examen abdominal rapportait une sensibilité hypogastrique sans masse palpable. L'examen pleuro-pulmonaire, neurologique et des aires ganglionnaires étaient normaux. Le reste de l'examen somatique était sans particularité. La patiente a bénéficié d'un curetage et une biopsie de la masse cervicale. L'étude histologique révélait une prolifération de cellules fusiformes peu différenciée dans un tissu conjonctif lâche (Figure 2). L'étude immuno-histochimique montrait la forte expression d'anticorps anti-desmine, d'anti-myogénine et anti-vimentine en faveur d'un rhabdomyosarcome embryonnaire botryoïde. L'IRM abdomino-pelvienne montrait une volumineuse masse du col utérin de 114*90*114 mm solido-kystique comblant la sub-totalité de la fossette pelvienne. Elle atteint l'endocol et l'exocol et s'étend au tiers supérieur du vagin. L'IRM note aussi l'absence d'extension au corps utérin, de métastases hépatiques, d'adénopathies abdomino-pelviennes et d'absence épanchement (Figure 3, Figure 4, Figure 5). La patiente présentait une insuffisance rénale obstructive pour laquelle elle a bénéficié d'une néphrotomie droite. Le scanner thoracique fait après montée sonde était normal. Après stabilisation de la fonction rénale, Une chimiothérapie pré-opératoire type VAC fut débutée. La patiente a reçu 5 cures de VAC avec une régression du processus tumoral de 90% au toucher vaginale et à la TDM abdomino-pelvienne par rapport à la taille initial. Patiente fut opérée et a bénéficiée d'une hystérectomie sans conservation des annexes. L'étude histo-pathologique de la pièce de résection montrait un remaniement fibreux inflammatoire du col utérin avec foyer résiduel tumoral de 5%, les paramètres et la collerette vaginal étaient sains. La malade a reçu par la suite 2 cures post-opératoires, puis elle a bénéficié d'une radiothérapie post-opératoire sur le pelvis.

## Discussion

Le rhabdomyosarcome du col est une tumeur rare, elle représente moins de 1% des cancers du col [1]. La localisation vaginale est 5 fois plus fréquente que la localisation cervicale, et se voit généralement chez des femmes en PAG ou chez des jeunes filles avec âge moyen entre 10 et 20 ans en fonction des séries [2]. Cette tumeur mésenchymateuse fait partie du groupe mésodermique [3]. Les circonstances habituelles de découverte sont l'apparition de métrorragie récidivantes associées ou pas à des leucorrhées. Au stade débutant, l'examen montre la présence d'un polype d'apparence bénin qui récidive rapidement après son ablation [3]. Ceci explique la difficulté du diagnostic précoce. A stade avancé de l'extension locale, le processus tumoral prend un aspect classique polyploïde dit en « grappe de raisin » [4]. Dans notre observation, la patiente rapportait des métrorragies abondantes révélant une tumeur en grappe de raisin après résection 10 mois auparavant d'un polype bénin. Les formes anatomo-pathologiques du rhabdomyosarcome sont, selon l'Intergroup Rhabdomyosarcoma Study (IRS), la Société Internationale d'Oncologie Pédiatrique (SIOP), le National Cancer Institue (NCI): le rhabdomyosarome anaplasique, embryonnaire et alvéolaire. Notons que le rhabdomyosarcome botryoide, décris pour la première fois en 1892 par Pfannestiel, fait partie du groupe de rhabdomyosarcome embryonnaire [2, 5]. L'étude histopathologique du type embryonnaire montre une prolifération de cellules rondes ou fusiformes au sein de laquelle se trouvent des cellules immatures de différenciation musculaire dites rhabdomyoblaste. Ces cellules sont à cytoplasme éosinophile avec un noyau rond et hyperchromatique associés à une double striation évoquant une origine musculaire. Ces cellules baignent dans un tissu conjonctif lâche œdémateux composé d'une trame claire et granuleuse [2, 6]. Le groupe embryonnaire comporte deux sous-groupes: le rhabdomyosarcome botryoïde et à cellule fusiforme [6]. L'immuno-marquage confirme l'origine musculaire par expression de la desmine, de l'actine et la présence d'immuno-histochimie par la myoglobine [7–10].

Dans notre observation, l'étude des marqueurs tumoraux tissulaires montraient une positivité à la desmine et à la myogénine. En termes de modalité d'extension, le rhabdomyosarcome du col est une tumeur à extension locorégionale massive avec des récidives fréquentes [3]. Les métastases sont rares [11, 12]. Le bilan d'extension ainsi préconisé, comportera un scanner pelvien ou IRM pelvien pour statuer sur l'extension locorégionale de la tumeur primitive [2]. Vu la rareté des métastases, on se contentera d'une échographie abdominale et une radiographie thoracique [2].

Le traitement local doit être ainsi agressif, initialement mutilant sanctionné par une exentération pelvienne. Il devient, grâce à la chimiothérapie néo-adjuvante, de plus en plus conservateur, allant d'une polypectomie à une hystercetomie, colpectomie ou une hyphadénectomie permettant ainsi dans certain cas la conservation de la fertilité chez la jeune fille [13, 14]. La chimiothérapie néo-adjuvante dans la plupart des cas rapporté par la littérature préconise les protocoles VAC (Vincristine-Actinomycine-Cyclophosphamide) ou VA (Vincristine-Actinomycine) pendant 6 à 12 cures précédant une chirurgie [14–16]. Notre patiente a reçu 5 cures pré-opératoire de type VAC. En per-opératoire, la tumeur était localisées sans extension locorégionale. La résection de la tumeur était complète. Ceci permettait de classer la tumeur selon la classification de l'Intergroup Rhabdomyosarcoma Study (IRS) en groupe 1. La classification de l'IRS comporte 4 groupes (Tableau 1). Quant à la radiothérapie, il n y a aucune preuve de son efficacité. Elle est indiquée en cas de résidu tumoral ou d'adénopathie pelvienne [17]. Notre patiente a bénéficié d'une radiothérapie après hystérectomie vu la présence d'une adénopathie nécrotique au scanner post-opératoire. Elle est contre-indiquée dans l'IRS [1, 14]. La survie à 5 ans est estimée à plus de 60% tous stades confondus et à plus de 90% pour une maladie localisée [16].

**Tableau 1:** Classification de l’Intergroup Rhabdomyosarcoma Study (IRS) en fonction de l’extension de la masse et la qualité de la résection

Groupe	Description
Groupe I	Tumeur localisée, ablation microscopique complète, confinée au muscle ou à l’organe d’origine sans envahissement ganglionnaire
Groupe II	Ablation macroscopique totale mais persistance d’un tissu tumoral microscopique. Maladie régionale mais complètement réséquée ou extension aux ganglions qui ont été complètement réséqués
Groupe III	Tumeur localisée, ablation microscopique complète, confinée au muscle ou à l’organe d’origine sans envahissement ganglionnaire.
Groupe IV	métastases à distance au diagnostic.

## Conclusion

Le RMS du col utérin est une tumeur rare qui survit essentiellement chez la jeune fille. L'extension est surtout locorégionale. Le traitement consiste en un geste chirurgical à minima associé à une chimiothérapie péri-opératoire. La place de la radiothérapie demeure imprécise.
